# Effects of an eHealth Cardiac Exercise Rehabilitation Platform for Patients After Percutaneous Coronary Intervention Based on the Persuasive Systems Design Model: Randomized Controlled Trial

**DOI:** 10.2196/71450

**Published:** 2026-01-14

**Authors:** Yang Liu, Xiting Huang, Ziying Dai, Zhili Jiang, Wenxiao Wu, Jing Wang, Zhiqian Wang, Luyao Yu, Hanyu Li, Lihua Huang

**Affiliations:** 1 Department of Nursing The First Affiliated Hospital Zhejiang University School of Medicine Hangzhou China; 2 Department of Nursing, Sun Yat-sen University Cancer Center Guangzhou China; 3 School of Humanities and Management, Zhejiang Chinese Medical University Hangzhou China

**Keywords:** coronary heart disease, percutaneous coronary intervention, PCI, exercise rehabilitation, persuasive systems design, eHealth

## Abstract

**Background:**

Cardiac exercise rehabilitation is an important intervention for disease management of patients with coronary heart disease (CHD) after percutaneous coronary intervention (PCI). Still, the participation and compliance with exercise rehabilitation remain suboptimal. Mobile health technology is a promising approach to promoting involvement in cardiac exercise rehabilitation. Remote rehabilitation can overcome the problems existing in traditional rehabilitation.

**Objective:**

This study aimed to evaluate the effects of an eHealth cardiac rehabilitation (CR) platform based on the persuasive systems design model in addition to standard CR after PCI on physical activity (PA), exercise endurance, self-perceived fatigue, exercise self-efficacy (ESE), and quality of life for patients after PCI.

**Methods:**

A single-blinded, parallel, randomized controlled trial design was used. The study was conducted in the Department of Cardiology of a tertiary hospital in Hangzhou, China. A total of 180 eligible patients with CHD were enrolled from June to December 2023. Participants were randomly assigned (1:1) to the intervention group or the control group, with 90 patients in each group. The study is a 24-week eHealth CR program. The primary outcome was PA level; the secondary outcomes included exercise endurance, self-perceived fatigue, ESE, and quality of life. Data on the primary and secondary outcome measures were collected at baseline (T0), at 12 weeks of intervention (T1), and at 4 (T2), 8 (T3), and 12 (T4) weeks of follow-up. The generalized estimating equation model was used to examine changes in the outcome variables between the 2 groups across the study end points.

**Results:**

Generalized estimating equation analyses revealed significant group-by-time interactions for all outcome measures (all *P*<.001). At T4, compared with the control group, the intervention group demonstrated statistically significant improvements in the following outcomes: PA: median 1723.00 versus 805.50 Metabolic Equivalent Task minutes per week (β coefficient=937.29, 95% CI 867.61-1006.97); 6-minute walk distance: median 436.00 versus 405.00 m (β coefficient=31.00); self-perceived fatigue: median 9.00 versus 10.00 (β coefficient=–1.00, indicating reduced fatigue); ESE: 61.11 versus 27.78 (β coefficient=33.33); Short Form of 36 Health Survey Questionnaire score: 91.19 versus 84.13 (β coefficient=7.06; all *P*<.001). Notably, there was no significant difference in self-perceived fatigue between the 2 groups at T1 (*P*=.50).

**Conclusions:**

The findings of this study demonstrate the effectiveness of the eHealth CR based on the persuasive systems design model in addition to standard CR after PCI in improving the PA level, exercise endurance, ESE, quality of life, and self-perceived fatigue of patients. These findings also provide insights into the application of an eHealth cardiac exercise rehabilitation interventions to enhance the rehabilitation of patients with CHD.

**Trial Registration:**

China Clinical Trials Registry (ChiCTR) ChiCTR2300071666; https://www.chictr.org.cn/showprojEN.html?proj=197908

## Introduction

### Background

Cardiovascular diseases (CVDs) have long been a global health scourge, exacting a heavy toll on both individual well-being and health care systems worldwide. The World Health Organization (WHO) highlights that CVDs claim approximately 17.9 million lives annually, constituting a staggering 32% of all global deaths [1]. In China, the situation is equally disconcerting. With the rapid pace of urbanization, changes in lifestyle, and an aging population, the prevalence of CVDs, especially coronary heart disease (CHD), has been on an upward trajectory [2,3]. This has led to a substantial increase in the number of patients undergoing percutaneous coronary intervention (PCI), a common and effective treatment for CHD.

Despite the remarkable advancements in PCI technology, which significantly improve the acute condition of patients by restoring blood flow to the heart, it is not a cure-all solution. Patients post PCI often face a plethora of challenges. Exercise intolerance is a prevalent issue, as a large proportion of these patients experience a decline in their physical capacity, which restricts their daily activities and quality of life [4]. Self-perceived fatigue is another common complaint, which can be attributed to the physiological stress of the intervention, underlying cardiac damage, and the body’s recovery process [5]. Moreover, many patients struggle with suboptimal exercise self-efficacy (ESE), lacking the confidence to engage in regular physical activity (PA), which is crucial for their long-term cardiac health [6].

Cardiac rehabilitation (CR) has emerged as an essential component of post-PCI care. It is a comprehensive, multidisciplinary program that encompasses exercise training, risk factor modification, psychological counseling, and patient education [7]. Extensive research has demonstrated its effectiveness in improving exercise capacity, reducing cardiovascular risk factors, enhancing psychological well-being, and ultimately decreasing mortality and morbidity rates among patients post PCI [8]. For instance, a network meta-analysis found that multiple exercise interventions enhanced peak oxygen consumption, with high-intensity interval training showing the greatest effect, followed by combined water-based and moderate-intensity continuous training, and other interventions like combined aerobic and resistance exercise also demonstrated benefits [9].

However, traditional CR programs, which are typically centered around in-hospital or clinic-based services, are plagued with limitations. Geographical barriers pose a significant challenge, especially for patients living in rural or remote areas, who may have to travel long distances to access these services [10]. Time constraints are another hurdle, as many patients, particularly those who are still working or have family responsibilities, find it difficult to fit regular rehabilitation sessions into their busy schedules. Additionally, the cost associated with in-person rehabilitation, including transportation and potential loss of income during treatment, can be a deterrent for some patients [11]. The proportion of patients participating in the CR program is only 25% to 35% [12].

In recent years, remote cardiac rehabilitation (RCR) has been advancing rapidly, and the potential exists for the challenges of traditional facility-based CR programs to be addressed by delivering care to patients in the convenience of their own homes with real-time, personalized support [11]. The RCR uses different methods such as the internet, wearable devices, and mobile apps [13]. Despite the positive outcomes observed in the application of digital health interventions for CR, such as SMS, remote electrocardiographic monitoring, and mobile or web portal tools, these advancements have predominantly remained in research settings and have not yet been adopted widely in clinical practice [14]. A systematic review by Duff et al [15] assessed the application of behavior change techniques (BCTs) in eHealth interventions designed to increase PA in CVD, and identified feedback and monitoring as the most common BCT category implemented in these interventions. Behavior change theory proponents note that these theories explain how behavior change happens [16]. However, information system developers often prioritize using BCTs over understanding the underlying theories [17]. Systems lacking a strong theoretical basis may have conflicting mechanisms, harming long-term effectiveness.

Such systems are designed to form, alter, or reinforce the attitudes, behaviors, or compliance of their users voluntarily. A key element in behavior and attitude change is persuasion. The persuasive systems design (PSD) model addresses this gap by guiding the analysis of the persuasion context, including recognizing the intent of the persuasion, understanding the persuasion event, and defining the strategies in use [18]. It describes how to inject persuasive characteristics into the system to stimulate behavior change in the design process. In the functional design of the persuasive system, the characteristics of the system are divided into main task support, dialogue support, system credibility support, and social support [19], and a total of 28 persuasive principles are included. It fully explains how the design principles are translated into software requirements and further manifests the system characteristics. The information system developed and designed under the guidance of the framework can motivate users to implement self-management and trigger health behavior change [20].

### Aims of This Research

In the context of post-PCI CR, there is a paucity of well-designed mobile health (mHealth) interventions that fully leverage the PSD model to comprehensively address patients’ multifaceted needs. Existing studies either focus on single-aspect interventions or fail to fully capitalize on the potential of the PSD model. Therefore, this study aimed to develop and evaluate an eHealth CR platform grounded in the PSD model in addition to standard CR for patients post PCI. We hypothesized that such a platform could effectively improve patients’ PA levels, exercise endurance, ESE, and quality of life, while reducing self-perceived fatigue.

## Methods

### Study Design

The study was a single-blinded, parallel, randomized controlled clinical trial. The study protocol complied with the Declaration of Helsinki. The report was in accordance with the CONSORT (Consolidated Standards of Reporting Trials) guidelines.

### Participants

Participants were recruited in the Department of Cardiology of a tertiary hospital in Hangzhou, a provincial capital city in eastern China, from June to December 2023.

The inclusion criteria were as follows: (1) patients were older than 18 years old; (2) met the WHO diagnostic criteria of CHD, underwent PCI with 1 or 2 stents at first time, and were diagnosed as low-risk factors according to the risk classification of exercise rehabilitation of CHD; (3) patients with a left ventricular ejection fraction of 50% to 70%; (4) patients with cardiac function grade Ⅰ or Ⅱ; (5) transradial or ulnar artery puncture; (6) patients and their families agreed and actively cooperated with the exercise rehabilitation treatment; and (7) no language disorder, could read and speak Chinese, had no prescribed PA restrictions. Participants were excluded from the study if they (1) had presenting with cardiogenic shock, severe arrhythmia and cardiac function grade Ⅲ or above; (2) had severe liver and kidney dysfunction (liver dysfunction of Child-Pugh class B or C; estimated glomerular filtration rate <30 ml/[min·1.73m^2^]), severe anemia (hemoglobin<60 g/L); (3) had severe pulmonary disease, such as chronic obstructive pulmonary disease, emphysema, bronchiectasis, and pulmonary heart disease; (4) had a history of falls within half a month, or were unable to exercise independently; (5) took antianxiety or depression drugs; (6) had previous history of cerebral hemorrhage; and (7) could not use WeChat (Tencent Holdings Limited) skillfully.

### Sample Size

The sample size was determined by PA level based on a pilot trial, in which the SD was 133.81, and the mean difference between the 2 groups was 131. A total of 150 participants were required to detect a difference between the 2 groups at a 5% (2-sided) significant level with a power of 80%. Considering the loss to follow-up, the sample size of each group was at least 90, and the total sample size was at least 180, allowing a 20% dropout.

### Randomization and Blinding

A total of 180 eligible participants were randomly assigned to the intervention or control group at a 1:1 ratio via block randomization (block size=4) to ensure balanced group sizes. The randomization was implemented by an independent statistician (not part of the core study team, nor involved in recruitment, assessment, or intervention) to avoid selection bias.

The statistician used SPSS (version 26.0; IBM Corp) to generate a random number sequence, pairing each number with a unique participant ID. Group assignment was predefined: even numbers for the intervention group and odd numbers for the control group. For allocation concealment, each ID, random number, and group assignment was sealed in a sequentially numbered opaque envelope, stored in a locked cabinet. Upon recruitment, the research coordinator, unaware of the sequence, retrieved the next envelope to inform the participant of their group.

Finally, 90 participants were allocated to each group. The intervention lasted for 12 weeks, with an additional 12-week follow-up period. Notably, an eHealth cardiac exercise rehabilitation based on the PSD model was provided in addition to standard CR; the volume and content of standard rehabilitation were consistent between the 2 groups to ensure that group differences in outcomes could be attributed to the eHealth platform intervention. Due to the distinguishable eHealth platform intervention, blinding of participants and intervention deliverers was unfeasible, but outcome assessors remained blinded. Thus, this was a single-blinded, randomized controlled trial.

### Interventions

Both groups received standard CR to isolate the effect of the additional mHealth intervention in the intervention group. Standard care included in-hospital education, exercise guidance, and follow-up support.

In in-hospital education, a 60-minute group session was conducted by a cardiac nurse, covering PCI postcare knowledge included medication adherence, dietary management, warning signs of adverse events (AEs), and basic exercise principles.

In exercise guidance, a 30-minute one-on-one session was conducted with a physiotherapist to teach low-to-moderate intensity aerobic exercises, including brisk walking, stationary cycling, and resistance training, such as hand grip exercises suitable for home practice, with a recommended weekly volume of 150 minutes of moderate-intensity activity.

In the follow-up support, monthly 15-20 minutes each telephone check-ins were conducted to address patient questions, remind them of exercise and medication adherence, and collect brief feedback on physical status.

### Intervention Group: Standard Care+mHealth Platform

#### Overview

**Figure 1:**
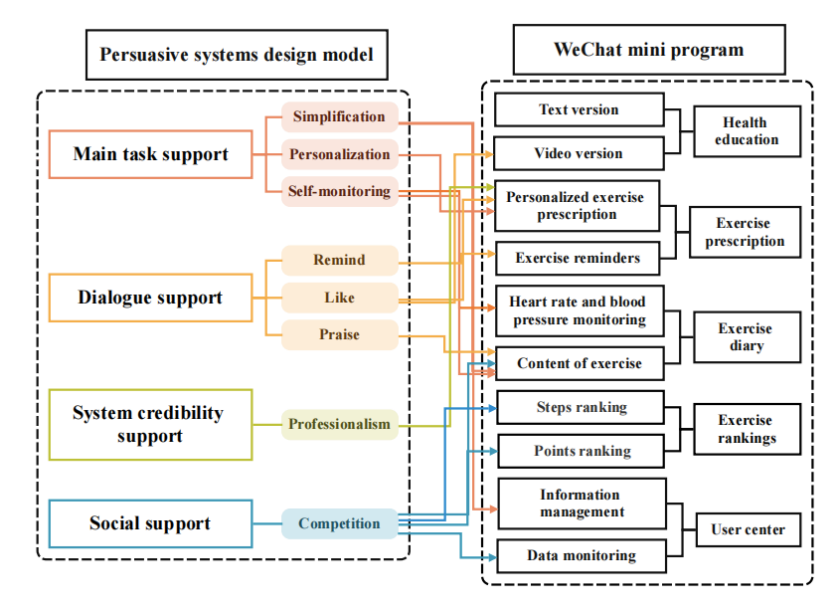
Schematic diagram of the persuasive systems design–based eHealth application development process for patients with coronary heart disease after percutaneous coronary intervention.

The intervention group received a PSD model–based eHealth exercise rehabilitation in addition to standard CR. We established a development team composed of cardiology experts, physiotherapists, clinical nursing experts, evidence-based nursing experts, software engineers, and artists. According to the design content, we constructed an exercise rehabilitation WeChat applet for patients with CHD after PCI. The specific construction process of the WeChat mini program is detailed in Figure 1.

The eHealth platform was developed exclusively under the guidance of the PSD model—a theoretical framework specifically tailored for designing digital tools to stimulate behavior change. The platform’s functional modules and design logic (schematic diagram illustrated in Figure 1) directly align with the 4 core components of the PSD model (main task support, dialogue support, system credibility support, and social support). The detailed integration of the PSD model into the intervention, its connection to global eHealth behavior change frameworks, and how it guided the platform’s design are described further in this study.

#### Main Task Support: Simplifying Exercise Rehabilitation Adherence

#### PSD Theoretical Basis

The PSD model defines “main task support” as designing features to reduce user effort in completing core goals via principles like simplification and personalization. This component addresses a key barrier to digital health adherence—complex or nontailored tasks that increase user burden.

#### Integration Into the Platform

### Simplification Principle

The home page was designed with a single “Exercise Prescription” button as the core entry point, reducing navigation steps from an average of 4 clicks to 1. Postexercise logging only requires 3 inputs (duration, heart rate, and discomfort), avoiding redundant data entry.

### Personalization Principle

Based on baseline 6-minute walk distance (6MWD), left ventricular ejection fraction, and exercise preferences, the platform automatically generates individualized exercise plans (eg, 30-minute brisk walking for patients with 6MWD > 350 m vs 20-minute slow walking for 6MWD < 300 m)—aligned with PSD’s focus on “matching tasks to user capabilities.” This personalization is distinct from generic plans in non-PSD eHealth tools, which often use fixed templates.

### Self-Monitoring Principle

Patients could record the data before and after each exercise in a diary, and track the progress of setting goals over time, so that patients could clearly understand their performance or status in the exercise rehabilitation process, and the rehabilitation platform can support patients to achieve self-reporting and motivate patients to achieve exercise goals.

#### Dialogue Support: Enhancing Real-Time Interaction and Feedback

#### PSD Theoretical Basis

“Dialogue support” in the PSD model refers to interactive features that guide behavior via reminders, praise, and preference matching—critical for maintaining user engagement in long-term rehabilitation.

#### Integration Into the Platform

### Reminder Principle

Patients set customizable exercise reminders (eg, “7 PM daily walk”) with pop-up and vibration alerts. The platform also sends adaptive reminders (eg, “You haven’t exercised in 3 days—start with a 15-minute walk today”) if adherence drops, addressing the “forgetfulness” barrier common in patients post PCI.

### Praise Principle

After each exercise session, the platform generates immediate positive feedback (eg, “Great job! You completed 100% of today’s plan”) and weekly “adherence badges” (eg, “3-week Consistent Exercise Award”). This aligns with PSD’s goal of reinforcing desired behaviors through positive reinforcement.

### Like Principle

Patients can also receive “likes” from the research team and anonymous peers (via the “Exercise Ranking” module) for completing exercise plans or achieving personal goals. The platform sends real-time notifications of likes (eg, “3 peers praised your consistent exercise!”), leveraging social approval to enhance motivation—consistent with PSD’s dialogue support, focus on interactive feedback.

#### System Credibility Support: Building Trust in Digital Guidance

#### PSD Theoretical Basis

“System credibility support” ensures users perceive the platform as reliable via professionalism and transparency—a prerequisite for accepting digital health advice, especially in cardiac care.

#### Integration Into the Platform: Professionalism Principle

All exercise prescriptions and educational content were co-developed by a team of 2 cardiologists and 1 CR nurse, with references to the guidelines of the European Society of Cardiology [21] displayed in the “Health Education” section.

#### Social Support: Leveraging Social Influence for Adherence

#### PSD Theoretical Basis

“Social support” in the PSD model uses competitive or cooperative features to motivate behavior via social comparison—proven effective in digital health interventions for exercise adherence.

#### Integration Into the Platform: Competitive Principle

The “Exercise Ranking” module displays anonymized weekly exercise completion rates of other participants (eg, “You are in the top 25% of users this week”)—without personal identifiers to protect privacy. This feature leverages mild social comparison to boost motivation, consistent with PSD’s focus on positive social influence [22] and avoiding the pressure of direct competition.

#### Iterative Phases

The PSD model–based eHealth platform was developed in 4 iterative phases, with pilot testing integrated to refine functionality:

#### Phase 1: Framework Design (Month 1-2)

Guided by the PSD model, we identified four core persuasive features for CR:

1. Primary task support: personalized exercise prescriptions aligned with frequency, intensity, time, and type principles to simplify task completion.

2. Dialogue support: real-time feedback and reminders to enhance user engagement, including praise, likes, and adaptive reminders.

3. Credibility support: evidence-based educational content cited from the guidelines of the European Society of Cardiology [21] to build trust.

4. Social support: using competitive features to motivate behavior, a multidisciplinary team collaborated to draft the platform’s functional modules (health education, exercise prescription, exercise diary, and exercise rankings) and user interface wireframes.

#### Phase 2: Prototype Development (Month 3-4)

Based on the framework, a prototype version (V1.0) was developed with basic functionalities—exercise plan generation based on baseline data input, manual heart rate logging (preliminary version before integrating wearable device connectivity), and static educational articles (n=10) on post-PCI care.

#### Phase 3: Pilot Testing and Revision (Month 5)

To validate usability and feasibility, we conducted a pilot test with 20 patients post PCI (mean age 62.3, SD 7.5 years; 12/20, 60% male) who met the study’s inclusion criteria.

#### Pilot Testing Procedures

Usability assessment: Patients used V1.0 for 4 weeks, then completed the System Usability Scale (SUS) [23].

Functional evaluation: The research team recorded technical issues, such as loading delays and calculation errors relating to exercise volume, as well as adherence metrics, such as the daily login and module completion rates.

#### Key Revisions After Pilot Testing

Technical fixes: In total, 2 critical bugs were resolved (eg, an incorrect MET [Metabolic Equivalent Task]-min/week calculation), and the loading speed was optimized by 40%.

Usability improvements: Simplified the exercise logging interface (reduced input fields from 5 to 3) and added video tutorials for first-time users.

Content expansion: Increased educational materials to 20 (added 5 videos on exercise form correction based on patient feedback).

The revised version (V2.0) achieved a posttest SUS score of 78.5 (SD 6.2) versus 62.3 (SD 8.1) for V1.0 (*P*<.01), indicating acceptable usability.

#### Phase 4: Finalization (Month 6)

Incorporated feedback from the pilot test to finalize the platform (V3.0), which included all features personalized, simplification, self-monitoring, and so on. A 1-day training session was conducted for researchers to ensure consistent data collection during the formal study.

#### Establishment of the Exercise Prescription Library

**Figure 2:**
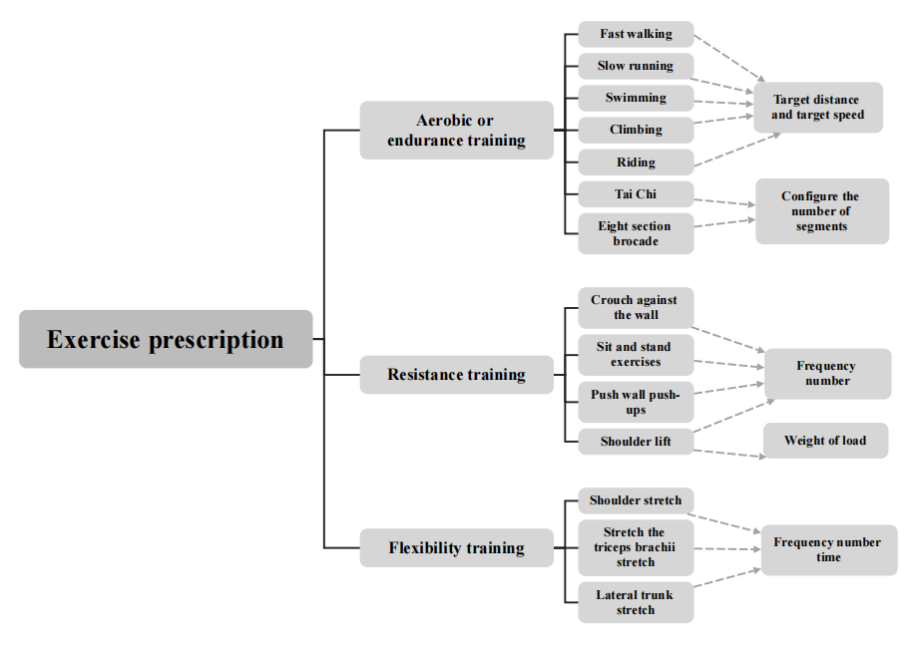
Exercise prescription library for cardiac rehabilitation in patients with coronary heart disease after percutaneous coronary intervention in a tertiary hospital in Hangzhou, China.

To ensure the safety, feasibility, and effectiveness of exercise interventions for patients post PCI, we developed a tailored exercise prescription library based on evidence summary and multidimensional considerations, as illustrated in Figure 2.

#### Evidence Summary for Prescription Development

First, we conducted a systematic review of guidelines and studies on post-PCI exercise rehabilitation, including (1) 2021 ESC Guidelines for CR [21], which recommend 150 minutes/week of moderate-intensity aerobic exercise for patients with CHD; (2) a network meta-analysis by Gomes-Neto et al [9], confirming that structured exercise improves exercise capacity in patients post PCI; and (3) Chinese expert consensus on CR [24], emphasizing gradual intensity progression and individualization for Chinese populations.

From these, we extracted core parameters, that is, suitable intensity (40-60% of maximal oxygen uptake), optimal frequency (3-5 sessions/week), and safe duration (20-40 minutes/session for moderate-intensity activity).

The details of the multidimensional considerations in library construction are presented in Multimedia Appendix 1.

The library was reviewed and approved by a panel of 3 CR specialists (with more than 10 years of experience) to ensure clinical appropriateness.

**Figure 3:**
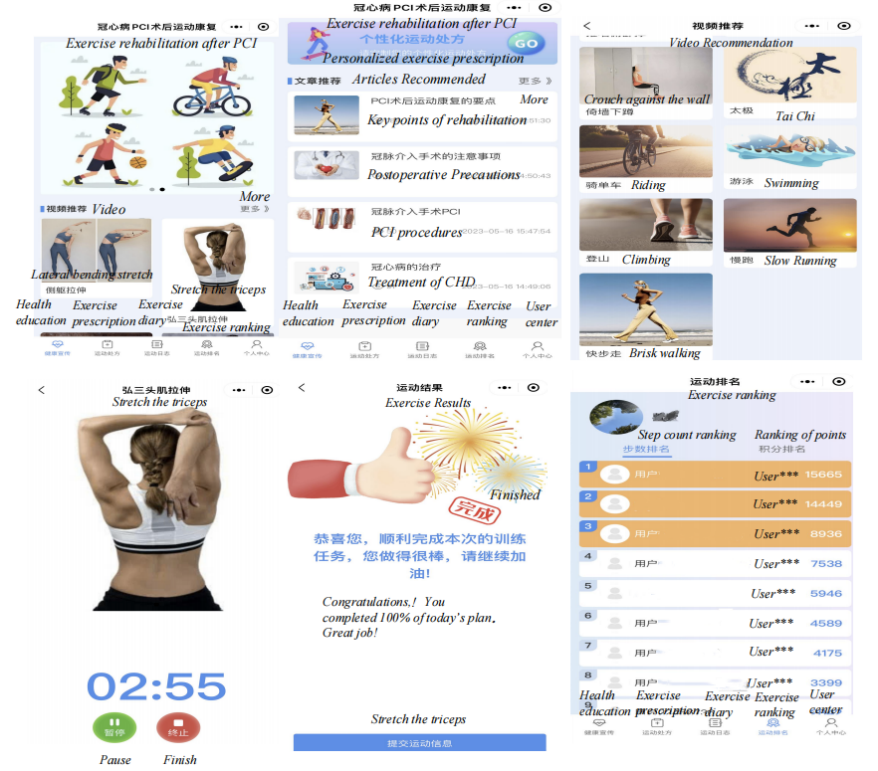
User interface diagram of the persuasive systems design–based WeChat mini program for exercise rehabilitation in patients with coronary heart disease after percutaneous coronary intervention.

There were 5 basic modules in the program, namely, health education, exercise prescription, exercise diary, exercise ranking, and personal center. The user interface design diagram is illustrated in Figure 3.

We introduced the use of a WeChat mini program to patients before discharge, ensuring that patients could successfully operate the mini program. The mini program’s health education module allows patients to access disease knowledge through text and video formats, complete cardiac function grading assessments, 6-minute walking tests, Borg fatigue ratings, and monitor relevant vital signs within 1 week after discharge. Personalized exercise prescriptions are then tailored for patients based on comprehensive data evaluation. Patients can set weekly exercise reminders and follow the guidance process of the mini program to start their exercise rehabilitation. After each session, vital signs and discomfort symptoms during exercise are recorded in the exercise log module. Medical staff can timely adjust patients’ exercise prescriptions according to their weekly situation and provide feedback on problems. Participants were required to continue to use the WeChat mini program for 12 weeks, and the step tracking function of the WeChat mini program was active during the 12-week intervention period. The duration of using the WeChat mini program, the frequency of weekly login to the platform, and the specific exercise data recorded in the exercise log could also be monitored through the data background of the medical and nursing side.

According to the frequency, intensity, time, and type principle, the specific structure of the selected exercise items, namely the frequency, intensity, and time of exercise, can be adjusted, which can realize the personalized adjustment of exercise tasks and task details, and provide a basis for the customization of personalized exercise prescription. Low-intensity exercise was defined as 30%-49% of maximal heart rate (or 3-5 METs), requiring aerobic or endurance training twice a week; moderate-intensity exercise as 50%-69% of maximal heart rate (or 6-8 METs), with aerobic or endurance training 3 times a week, resistance training once a week, and flexibility training once a week. High-intensity exercise, as ≥70% of maximal heart rate (or ≥9 METs), consisted of aerobic or endurance training 5 times a week, resistance training twice a week, and flexibility training twice a week.

#### Measurements

#### Overview

A self-designed demographic questionnaire was used to collect baseline sociodemographic data, including age, sex, marital status, education, employment status, living conditions, course of disease, and so on. Clinical data, including comorbidities, number of diseased vessels, cardiac function classification, PA level, scores of exercise endurance, self-perceived fatigue, ESE, and quality of life, were retrieved from the medical records. Primary and secondary outcomes were assessed with validated questionnaires. Questionnaires were collected by trained researchers through a pencil-and-paper survey. A previous intervention study [25] suggested that the exercise rehabilitation effect of patients with CHD after PCI should be intervened for at least 12 weeks, and the difference was significant. Therefore, the intervention period of this study was 12 weeks, and follow-up was conducted at 4 weeks, 8 weeks, and 12 weeks after the end of the intervention. Data collection time points were: within 3 days of admission (T0), at 12 weeks of intervention (T1), 4 weeks of follow-up (T2), 8 weeks of follow-up (T3), and 12 weeks of follow-up (T4).

#### Primary Outcome

The primary outcome focused on increased PA, which was assessed at baseline (T0), at 12 weeks of intervention (T1), 4 weeks of follow-up (T2), 8 weeks of follow-up (T3), and 12 weeks of follow-up (T4). PA comprises body movements that use energy. PA level was defined as weekly exercise volume, quantified by MET-min/week via the International Physical Activity Questionnaire (IPAQ; Long Form) [26]. The 27-item IPAQ-Long Form evaluates 4 domains (walking, moderate- to vigorous-intensity activity, and sitting time). Participants reported activity frequency (days/week) and duration (minutes/day), which was converted to MET-min/week using standard values (3.5 METs for walking, 4.0 for moderate, and 8.0 for vigorous activity). It has cross-cultural reliability (test-retest *r*=0.75) and validity across 12 countries. The Chinese version showed internal consistency (Cronbach α=0.78) and criterion validity (*r*=0.42 with accelerometer data, *P*<.01), suitable for Chinese patients post PCI [27].

#### Secondary Outcomes

The secondary outcomes focused on exercise endurance, self-perceived fatigue, ESE, and quality of life, which were also measured at baseline (T0), at 12 weeks of intervention (T1), and at 4 weeks of follow-up (T2), 8 weeks of follow-up (T3), and 12 weeks of follow-up (T4) weeks of follow-up.

#### Exercise Endurance

Exercise endurance was measured by the 6-minute walk test (6MWT), a standardized field test originally developed by Guyatt et al [28] to assess functional exercise capacity in patients with chronic respiratory or cardiac problems. Following American Thoracic Society guidelines [29], participants walked back-and-forth along a 30 m flat corridor (red turn markers); researchers instructed “fastest comfortable pace” and gave standardized encouragement every 1 minute. The total 6MWD was recorded. A portable pulse oximeter monitored peripheral oxygen saturation (SPO₂)—test paused or terminated if SPO_2_<88% or severe discomfort occurred. The 6MWT correlates with maximal oxygen uptake (*r*=0.6-0.8) in patients with cardiac problems [29].

#### Self-Perceived Fatigue

Self-perceived fatigue was assessed using the Borg fatigue rating scale. According to the Borg fatigue rating scale, 6-8 points indicate very very easy, 9-10 points indicate very easy, 11-12 points indicate easy, 13-14 points indicate slight exertion, 15-16 points indicate exertion, 17-18 points indicate very exerted, and 19-20 points indicate very very exerted [30].

#### ESE

Measured with the Exercise Self-Efficacy Scale (ESES), developed by Resnick and Jenkins [31] for older adults and validated in CR. The 10-item scale assesses confidence in exercise tasks, such as “walking 1 km” (1=“not at all confident” to 10=“completely confident”). Total score=sum of items; higher scores=greater self-efficacy. The ESES had passed the reliability and validity test, with a Cronbach α of 0.92. The reliability coefficients of each dimension ranged from 0.72 to 0.91, indicating good reliability. The structural validity of factor analysis showed that the cumulative variance of the 4 factors was 70.19%, and the factor load of each item was 0.606-0.909, indicating good validity [31].

#### Quality of Life

Quality of life was assessed using the Short Form of 36 Health Survey Questionnaire (SF-36), which evaluates the influence of CHD on individuals’ physical, emotional, and social well-being. The SF-36 questionnaire has a total of 8 dimensions and 36 items. Except for physiological function and emotional function, which are answered with “yes” and “no,” the other items are scored according to 3-6 levels, and the scores of each dimension are finally converted to 0-100 points. Cronbach α coefficient of the total volume table is 0.91. The scale has been validated in Chinese populations, demonstrating good reliability and validity [32].

#### Usability Indicator

Usability evaluation forms an integral part of the electronic product development process. It acts as a crucial factor in ensuring the successful implementation of telemedicine programs and provides a vital means to promote user acceptance and improve compliance among users [33]. System usability was measured at 12 weeks of intervention (T1) using the SUS. The 10-item scale includes 5 positive (eg, “I would like to use this system frequently”) and 5 negative (eg, “I found the system unnecessarily complex”) statements; 5-point Likert scale (1=“strongly disagree” to 5=“strongly agree”) [34]. Scoring criteria: (1) for odd-numbered items (1, 3, 5, 7, and 9): assign a score equal to the raw item score (ie, score= raw score); (2) for even-numbered items (2, 4, 6, 8, and 10): reverse score by calculating 5 minus the raw item score (ie, score=5–raw score). Compute the total sum of all item scores, then multiply by 2.5 to obtain the final score (sum×2.5=final score; range 0-100). Higher final scores indicate better usability of the platform. A score above 68 points is considered to be above average, a score below 68 is considered below average [23].

#### Safety Indicator

The incidence of AEs was used to evaluate the exercise safety of patients during the whole study period. If the patient has chest tightness, chest pain, arrhythmia, pale face, dizziness, gait instability, soft tissue injury, dislocation of bone and joint, fracture, and other conditions during exercise, the occurrence of one or more symptoms is considered as an AE, and the incidence of AEs is as follows: (Number of AEs÷total number of exercises)×100%. If there are adverse reactions, stop exercising immediately and seek help from medical personnel in time. The cause of the event will be analyzed by the research team, and all AEs will be reported to the Ethics Committee as required.

#### Statistical Analysis

Data analysis was performed using SPSS (version. 26.0), with 2-sided *P*<.05 considered statistically significant. An intention-to-treat approach was used. All randomly assigned participants (n=180, 90 per group) were included in the analysis, regardless of whether they completed the intervention or withdrew from follow-up. Missing data were handled via the generalized estimating equation (GEE) model, which inherently accounts for longitudinal data dependency and preserves the intention-to-treat principle. Descriptive statistics were used to summarize the participants’ characteristics—continuous variables as mean (SD) or median (IQR; Shapiro-Wilk test for normality) and categorical variables as n (%). Baseline between-group comparisons used independent samples *t* test (normal continuous data), chi-square test (categorical data), or Mann-Whitney *U* test (nonnormal continuous data); intragroup nonnormal data comparisons used paired Wilcoxon rank sum test. The GEE model analyzed all primary and secondary outcomes across T0-T4, adjusting for baseline outcome values, age, sex, and education level with an exchangeable correlation structure. Given the nonnormal distribution of outcome data (confirmed via the Shapiro-Wilk test), we initially considered distributional assumptions for skewed continuous data. However, after sensitivity analysis, a Gaussian distribution with an identity link function was ultimately selected for all outcomes (PA, 6MWD, ESES, SF-36, and Borg fatigue scale). This choice was justified by two key considerations. First, the large sample size (n=180) provides robustness to violations of normality via the central limit theorem; and second, the identity link yields interpretable additive β coefficients (directly reflecting between-group mean differences), which align with clinical relevance for rehabilitation outcomes (eg, absolute changes in MET-min/week or meters). The reference category was defined as “baseline (T0)+control group” for clear effect interpretation [35]. Bonferroni correction adjusted for multiple comparisons to ensure rigor.

#### Ethical Considerations

This study was conducted in compliance with the Declaration of Helsinki and approved by the Hospital Institutional Review Board (approval IIT20230069B-R2). Written, fully informed consent was obtained from all participants before their enrollment, detailing the study purpose, procedures, potential risks, and benefits. Participants retained the right to withdraw from the study at any time without penalty or impact on their subsequent medical care.

All participant data were managed in strict adherence to privacy and confidentiality protocols. Identifiable personal information was deidentified during data processing and storage, and access to the dataset was restricted to authorized research personnel only.

Participants were provided with a digital health management tool as compensation for their time and participation. The compensation arrangement was fully disclosed in the informed consent document, and participants voluntarily agreed to the terms before study participation.

## Results

### Patient Flow and Baseline Characteristics

**Figure 4:**
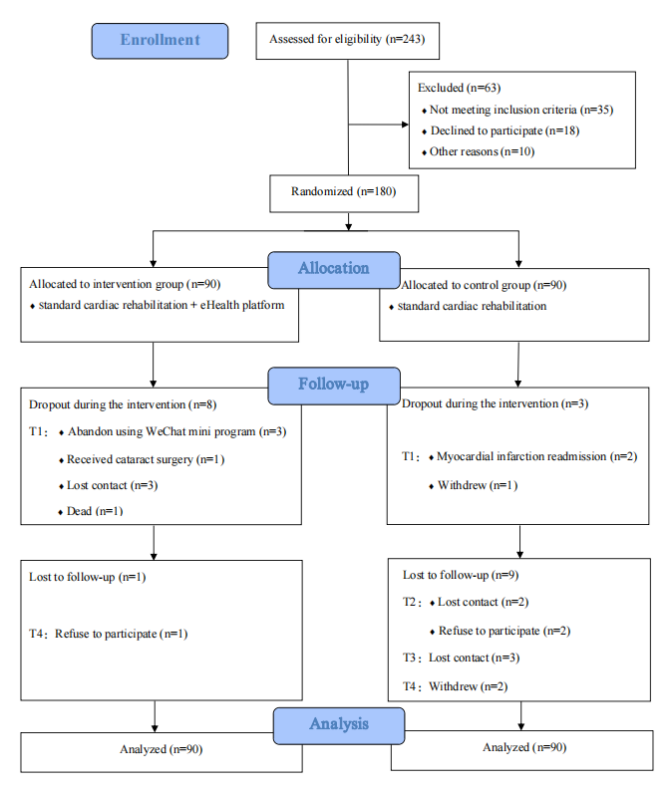
CONSORT (Consolidated Standards of Reporting Trials) flow diagram of the randomized controlled trial involving patients with coronary heart disease after percutaneous coronary intervention over the 24-week study period (12-week intervention+12-week follow-up) in Hangzhou, China. Analysis was conducted using an intention-to-treat approach, including all 180 randomly assigned participants regardless of intervention completion or follow-up status. Missing data were handled via the generalized estimating equation model.

A total of 180 participants were randomly allocated to the intervention group (n=90) or control group (n=90). Overall, 159 (88.3%) participants completed the follow-up. The CONSORT flow diagram is presented in Figure 4. As illustrated in Table 1, no statistically significant differences were observed between the intervention group and control group in all baseline characteristics (*P*>.05), including sociodemographic variables (age: mean 63.86, SD 9.17 y vs mean 62.68, SD 8.58 y; *P*=.21; and sex (male): 73/90, 81.1% vs 67/90, 74.4%; *P*=.28), clinical indicators (cardiac function grade I: 46/90, 51.1% vs 38/90, 42.2%; *P*=.23), and baseline values of outcome measures (6MWD: median 383.50, IQR 370.00-405.00 m vs median 380.00, IQR 373.00-386.00 m; *P*=.16; and PA: median 693.00, IQR 396.00-1031.25 MET-min/week vs median 648.00, IQR 495.00-1041.75 MET-min/week; *P*=.60). Consistent with the CONSORT guidelines, this baseline balance indicates that the randomization process was effective, and any subsequent differences in outcome measures between the 2 groups can be attributed to the intervention rather than preexisting group disparities.

**Table 1:** Baseline demographic, clinical characteristics, and outcome variables of patients with coronary heart disease after percutaneous coronary intervention in the intervention and control groups (Hangzhou, China).

Variable		Control group (n=90)	Intervention group (n=90)	*t* test (*df*), Chi-square (*df*), or *z* score		*P* value	
Age (y), mean (SD)		63.86 (9.17)	62.68 (8.58)	–1.266 (157)^a^		.21^b^	
BMI (kg/m^2^), mean (SD)		23.85 (2.37)	24.35 (2.07)	–1.425 (157)^a^		.16^b^	
**Sex, n (%)**					1.2 (1)^c^		.28^d^
	Male	73 (81.1)	67 (74.4)				
	Female	17 (18.9)	23 (25.6)				
**Marital status, n (%)**					—^e^		.12^f^
	Married	88 (97.8)	87 (96.7)				
	Unmarried	2 (2.2)	0 (0)				
	Widowed	0 (0)	3 (3.3)				
**Residence, n (%)**					2.3 (1)^c^		.13^d^
	Town	31 (34.4)	41 (45.6)				
	Countryside	59 (65.6)	49 (54.4)				
**Education level, n (%)**					0.5 (1)^c^		.98^d^
	Primary school or below	40 (44.4)	38 (42.2)				
	Junior high school	28 (31.1)	30 (33.3)				
	High school or technical secondary school	9 (10)	11 (12.2)				
	Junior college	7 (7.8)	6 (6.7)				
	Undergraduate or above	6 (6.7)	5 (5.6)				
**Professional types, n (%)**					3.7 (4)^c^		.46^d^
	Enterprises or institutions	7 (7.8)	2 (2.2)				
	Farmer or worker	36 (40)	38 (42.2)				
	Freelance	9 (10)	13 (14.4)				
	Retired	32 (35.5)	32 (35.6)				
	Unemployed	6 (6.7)	5 (5.6)				
**Health insurance, n (%)**					—		.44^f^
	Medical insurance for employees	23 (25.6)	25 (27.8)				
	Medical insurance for urban and rural residents	66 (73.3)	61 (67.8)				
	Other payment methods	1 (1.1)	4 (4.4)				
**Duration of illness (y), n (%)**					0.8 (1)^c^		.93^d^
	≤1	54 (60)	55 (61.1)				
	1-3	19 (21.1)	18 (20)				
	3-5	4 (4.4)	6 (6.7)				
	5-10	7 (7.8)	7 (7.8)				
	>10	6 (6.7)	4 (4.4)				
**Monthly income (¥; ¥1=US $0.14), n (%)**					1.6 (2)^c^		.46^d^
	≤3000	22 (24.4)	20 (22.2)				
	3001-4999	38 (42.2)	32 (35.6)				
	≥5000	30 (33.3)	38 (42.2)				
**Smoking** **, n (%)**					2.0 (2)^c^		.37^d^
	Yes	25 (27.8)	22 (24.4)				
	Ever	48 (53.3)	43 (47.8)				
	No	17 (18.9)	25 (27.8)				
**Drinking** **, n (%)**					2.4 (2)^c^		.30^d^
	Yes	8 (8.9)	15 (16.7)				
	Ever	59 (65.6)	54 (60)				
	No	23 (25.6)	21 (23.3)				
**Comorbidities, n (%)**					6.2 (4)^c^		.18^d^
	Hypertension	64 (71.1)	62 (68.9)				
	Diabetes	31 (34.4)	33 (36.7)				
	Dyslipidemia	5 (5.6)	17 (18.9)				
	Others	67 (74.4)	71 (78.9)				
	No	5 (5.6)	7 (7.8)				
**Blocked vessels, n (%)**					0.4 (1)^c^		.54^d^
	Single	13 (14.4)	16 (17.8)				
	Multiple	77 (85.6)	74 (82.2)				
**Cardiac function grade, n (%)**					1.4 (1)^c^		.23^d^
	I	46 (51.1)	38 (42.2)				
	II	44 (48.9)	52 (57.8)				
6MWD^g^, median (IQR)		383.50 (370.00-405.00)	380.00 (373.00-386.00)	–1.412^h^		.16^i^	
Borg fatigue rating, median (IQR)		12.00 (10.00-12.00)	12.00 (10.75-13.00)	–1.376^h^		.17^i^	
ESES^j^, median (IQR)		25.00 (22.22-27.78)	25.00 (22.22-31.25)	–0.173^h^		.86^i^	
PA^k^, median (IQR)		693.00 (396.00-1031.25)	648.00 (495.00-1041.75)	–0.522^h^		.60^i^	
SF-36^l^, median (IQR)		57.13 (53.44-67.09)	60.09 (50.94-66.72)	–0.963^h^		.34^i^	

^a^*t* test.

^b^Independent samples *t* test.

^c^Chi-square.

^d^Pearson chi-square.

^e^Not applicable.

^f^Fisher exact test.

^g^6MWD: 6-minute walk distance.

^h^*z* score.

^i^Mann-Whitney *U* test.

^j^ESES: Exercise Self-Efficacy Scale.

^k^PA: physical activity.

^l^SF-36: Short Form of 36 Health Survey Questionnaire.

### Primary Outcome

**Figure 5:**
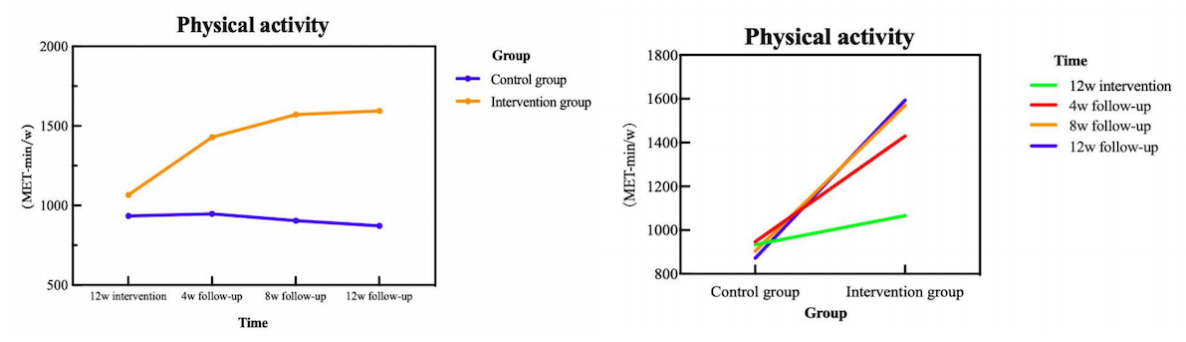
Changes in physical activity levels (MET-min/week) over the 24-week study period in patients with coronary heart disease after percutaneous coronary intervention.

The primary outcome, PA level, was demonstrated in Figure 5 and Table 2. For the primary outcome (PA level), GEE analysis was performed with baseline (T0)+control group as the reference category to clarify group differences and time-dependent intervention effects. The model adjusted for baseline PA level and accounted for the correlation of repeated measurements within participants using an exchangeable correlation structure. Baseline PA levels were balanced between groups in unadjusted analysis (Table 1; *P*=.60), confirming effective randomization. The GEE model’s “group (baseline, adjusted)” effect (β coefficient=48.254; *P*=.002) reflects the residual group difference after adjusting for age, sex, and education level—not an inherent baseline imbalance. This adjusted effect does not undermine randomization validity but enhances the model’s precision by accounting for potential confounding. The control group showed a modest natural increase in PA over time (eg, T4 vs T0: β coefficient=125.358, 95% CI 101.798-148.918; *P*<.001). In contrast, the intervention group exhibited significant additional improvements at all postbaseline time points, with the strongest effect at T4 (group×time interaction: β coefficient=937.288, 95% CI 867.609-1006.967; *P*<.001). The consistent significance of group×time interactions (all *P*<.001) confirmed that the intervention’s PA-enhancing effect was maintained throughout the study period.

**Table 2:** Generalized estimating equation analysis for physical activity levels (MET-min/week) over the 24-week study period in patients with coronary heart disease after percutaneous coronary intervention.

Parameter category^a^ (main effects) and parameter description		β coefficient (95% CI)	SE	*P* value
**Time (control group)**				
	T1 (12-week intervention) vs T0	23.521 (–0.149 to 47.191)	11.683	.05
	T2 (4-week follow-up) vs T0	46.857 (23.819 to 69.895)	11.748	<.001
	T3 (8-week follow-up) vs T0	31.523 (8.412 to 54.634)	11.809	.008
	T4 (12-week follow-up) vs T0	125.358 (101.798 to 148.918)	12.032	<.001
**Group (baseline, adjusted)**				
	Intervention Group vs Control Group (T0, adjusted for covariates)	48.254 (18.021 to 78.487)	15.389	.002
**Group×time interaction^b^ (intervention additional effect)**				
	T1 (12-week intervention)	861.109 (783.934 to 938.284)	39.376	<.001
	T2 (4-week follow-up)	899.386 (826.259 to 972.514)	37.311	<.001
	T3 (8-week follow-up)	879.404 (811.213 to 947.596)	34.792	<.001
	T4 (12-week follow-up)	937.288 (867.609 to 1006.967)	35.551	<.001

^a^Reference category: baseline (T0)+control group.

^b^β coefficients for group×time interactions represent the additional intervention effect (beyond the control group’s natural change) in the intervention group at each time point relative to baseline.

The GEE model used a Gaussian distribution with an identity link function, yielding additive β coefficients (absolute between-group differences). “Group (baseline, adjusted)” represents the adjusted group difference in PA at T0 after accounting for covariates (age, sex, and education level). This adjusted effect does not contradict the unadjusted baseline balance (Table 1, *P*=.60), as it reflects the residual group difference after covariate adjustment (a common observation in longitudinal models) and confirms no failure of randomization.

### Secondary Outcomes

Secondary outcomes are illustrated in Table 3. GEE analysis confirmed significant group-time interaction effects (all *P*<.001). Compared with the control group, the intervention group had significantly longer 6MWD at all time points (eg, T4: β coefficient=45.278, 95% CI 41.084-49.472; *P*<.001), lower self-perceived fatigue from T2 onwards (T4: β coefficient=–1.058, 95% CI –1.243 to –0.873; *P*<.001), and higher ESE (T4: β coefficient=31.015, 95% CI 29.019-33.010; *P*<.001) and quality of life (T4: β coefficient=6.857, 95% CI 6.171-7.543; *P*<.001) across all postbaseline assessments. No significant difference in self-perceived fatigue was observed at T1 (β coefficient=0.062, 95% CI –0.116 to 0.240; *P*=.50).

**Table 3:** Effects of the persuasive systems design–based exercise rehabilitation platform on secondary outcomes in patients with coronary heart disease after percutaneous coronary intervention over the 24-week study period.

Outcome measures		Control group, median (IQR)	Intervention group, median (IQR)	GEE^a^ β coefficient^b^ (95% CI)	*P* value
**6MWD^c^**					
	12 weeks of intervention	410.00 (389.00-420.00)	435.00 (423.00-448.00)	33.760 (29.311 to 38.208)	<.001
	4 weeks of follow-up	413.00 (395.00-421.00)	440.00 (430.00-448.00)	37.577 (33.346 to 41.808	<.001
	8 weeks of follow-up	406.00 (385.25-416.00)	439.50 (431.50-448.25)	44.495 (40.334 to 48.656)	<.001
	12 weeks of follow-up	405.00 (385.00-413.50)	436.00 (429.00-449.00)	45.278 (41.084 to 49.472)	<.001
**Borg fatigue rating**					
	12 weeks of intervention	10.00 (9.00-11.00)	10.00 (10.00-11.00)	0.062 (–0.116 to 0.240)	0.50
	4 weeks of follow-up	10.00 (9.00-10.00)	9.50 (9.00-10.00)	–0.331 (–0.512 to –0.150)	<.001
	8 weeks of follow-up	10.00 (9.00-10.00)	9.00 (9.00-9.00)	–0.707 (–0.881 to –0.533)	<.001
	12 weeks of follow-up	10.00 (9.00-10.00)	9.00 (8.00-9.00)	–1.058 (–1.243 to –0.873)	<.001
**ESES^d^**					
	12 weeks of intervention	33.33 (27.78-33.33)	58.33 (50.00-66.67)	23.355 (20.865 to 25.845)	<.001
	4 weeks of follow-up	30.56 (25.00-30.56)	61.11 (52.78-66.67)	28.865 (26.534 to 31.195)	<.001
	8 weeks of follow-up	30.56 (25.00-33.33)	61.11 (54.87-66.67)	28.012 (25.965 to 30.060)	<.001
	12 weeks of follow-up	27.78 (25.00-30.56)	61.11 (54.17-66.67)	31.015 (29.019 to 33.010)	<.001
**SF-36^e^**					
	12 weeks of intervention	80.19 (77.58-81.88)	89.44 (87.84-90.69)	9.520 (8.732 to 10.308)	<.001
	4 weeks of follow-up	81.13 (78.71-83.00)	90.56 (89.31-91.31)	9.443 (8.733 to 10.153)	<.001
	8 weeks of follow-up	82.56 (80.75-85.05)	90.88 (89.94-91.55)	8.138 (7.390 to 8.885)	<.001
	12 weeks of follow-up	84.13 (82.38-86.63)	91.19 (90.41-91.81)	6.857 (6.171 to 7.543)	<.001

^a^GEE: generalized estimating equation.

^b^β coefficients represent the estimated difference in outcome values between the intervention and control groups (positive β=higher outcome in the intervention group and negative β=lower outcome in the intervention group). All generalized estimating equation models were adjusted for baseline values of the respective outcome and sociodemographic covariates (age, sex, and education level).

^c^6MWD: 6-minute walk distance.

^d^ESES: Exercise Self-efficacy Scale.

^e^SF-36: Short Form of 36 Health Survey Questionnaire.

### Usability Indicator

**Figure 6:**
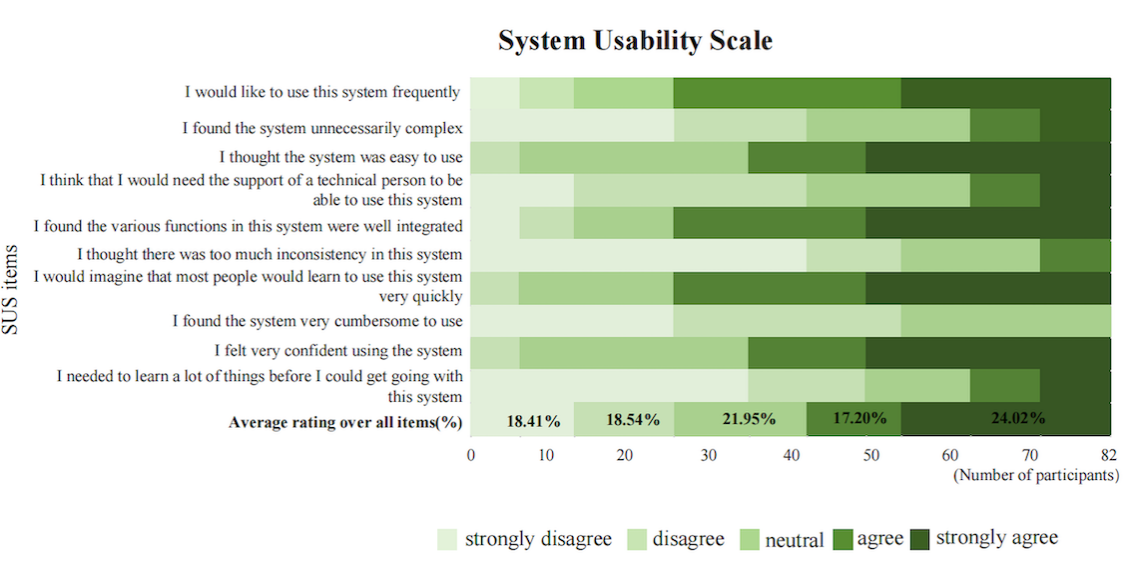
Summary plot of the System Usability Scale scores for the persuasive systems design–based WeChat mini program in patients with coronary heart disease after percutaneous coronary intervention (12-week intervention period). SUS: System Usability Scale.

The average SUS score of the WeChat mini program was 85.72 (SD 4.26) points (the highest score was 92 and the lowest score was 74.6, n=82), and its usability score was higher than the average level (≥68), indicating that the usability, ease of use, and learnability of the system were acceptable. The usability scores for the 10 SUS items and the average ratings for all items are summarized in Figure 6. Lighter shades of green indicate negative responses to the items (eg, “strongly disagree” for negative items and “strongly agree” for positive items), while darker shades of green indicate positive responses to the items (eg, “strongly agree” for negative items and “strongly disagree” for positive items).

### Safety Indicator

AEs during the study period were recorded comprehensively. As illustrated in Figure 4, three serious AEs were reported—2 cases of myocardial infarction readmission in the control group and 1 death in the intervention group. The 2 readmissions were unrelated to standard CR, and the death (acute cerebral hemorrhage in an 82-year-old male with hypertension and diabetes) was attributed to underlying comorbidities, not the exercise intervention. No other AEs (eg, chest tightness and musculoskeletal injury) occurred in either group. Mild muscle soreness in the early intervention stage resolved spontaneously. All serious AEs were documented, reported to the Ethics Committee, and managed per protocol.

## Discussion

### Principal Findings

This study confirmed that the PSD model–based mHealth platform provides added value beyond standard CR: both groups received consistent usual care, but the intervention group showed greater improvements in PA, exercise endurance, and ESE.

A key strength is the consistent delivery of standard care across both groups, which eliminated confounding from unequal usual care and clearly isolated the effect of the mHealth intervention. Our exclusive use of the PSD model addresses a key gap in CR eHealth studies. The platform’s strength lies in strict alignment between PSD principles and every functional module, rather than combining multiple theories such as social cognitive theory, self-efficacy theory, theory of planned behavior, and control theory [17]. For example, our platform embeds persuasive principles within intervention designs, ensuring theoretical consistency. This focused approach avoids the “theoretical fragmentation” common in multitheory interventions. Globally, RCR interventions primarily fall into 3 categories. Basic telemonitoring systems rely on wearable devices (eg, Google Fitbit or Apple Watch) to track steps and heart rate. These systems provide limited active guidance, focusing more on data collection than behavior change [36]. App-based educational tools provide static exercise videos and reminders, but they lack personalization and include fixed exercise plans regardless of the patient’s functional capacity [37]. Telehealth with human coaching combines weekly video consultations with physiotherapists and basic app tracking, but it is resource-intensive, limiting scalability [38]. In contrast, our intervention integrates PSD model–driven persuasive design with dynamic personalization—a gap in most RCR tools. Unlike basic telemonitoring or static apps, our WeChat mini program uses PSD-derived BCTs (self-monitoring via exercise diaries, social support via exercise ranking, reminders, and praise feedback) to actively motivate adherence.

The findings of PA are consistent with a previous study by Sankaran et al [39], which selected personalized and persuasive design principles to develop the HeartHab, a smartphone-based mobile app (developed by Hasselt University, Diepenbeek, Belgium) for telerehabilitation, by incorporating novel persuasive techniques to motivate patients with CHD to reach personalized PA targets. Compared with conventional rehabilitation, the effect of telerehabilitation treatment decreased at a lower rate after completion. A systematic review by Aldenaini et al [40] evaluated the effectiveness of persuasive techniques used to promote PA and reduce sedentary behavior and found that mobile and handheld devices were the most commonly used platforms, while the persuasive strategies most commonly used to achieve desired behavioral outcomes in the PA domain were self-monitoring and reminder. Salvi et al [41] found that by using the HeartCycle mobile medical system (a remote cardiovascular monitoring system developed by the research team of the University of Coimbra, Coimbra, Portugal; under a European Union–funded project coordinated by Philips, with participation of 17 partners including communication enterprises, hospitals, and universities, eg, Jessa Hospital, Hasselt, Belgium) to guide exercise, taking into account the design principles of the persuasive system and incorporating the principles of reward, reminder, suggestion, and rehearsal into the implementation process of the system, the exercise monitoring, guidance, and feedback are provided to patients to motivate them to persist in completing the rehabilitation plan, and the exercise time of patients is significantly increased. Exercise habits also improved after 6 months of follow-up, with high user acceptance and perceived usefulness. Psychological research shows that effective implementation of the behavior change intervention helps habit formation and helps for a long time to maintain motivation [42-44]. Moreover, when it comes to technology-supported interventions, it is important to design user-friendly and accessible systems. By considering the needs and perspectives of the user to enhance the user experience, human-computer interaction research has mainly focused on usability and interaction techniques when designing technology-supported rehabilitation systems. Consider a patient’s specific needs and ideas to help adjust these interventions based on technology, to accurately meet the target user, and to minimize the loss [42,45].

The 6MWT is a safe and effective tool to test exercise endurance, which has been widely used in clinical trials and the outcome measurement of cardiopulmonary rehabilitation [46]. The 6MWD was the main outcome index of the 6MWT. The study by Taylor et al [47] proposed that a within-group 6MWD improvement of at least 54 m constitutes a minimum clinically important difference (MCID) for patients with cardiac disease, reflecting meaningful functional recovery. It is important to distinguish this within-group change from the net intervention effect (between-group difference) reported in our study. For the intervention group, the within-group improvement in 6MWD from baseline (T0: 380.00 m) to T4 (436.00 m) was 56.00 m, exceeding the 54 m MCID threshold and indicating clinically meaningful functional gain for individual patients; the GEE β coefficients in Table 3 represent the net intervention effect (additional benefit of the PSD-based platform beyond the control group’s natural recovery), which ranged from 33.76 m (T1) to 45.28 m (T4). While this net effect did not reach the 54 m MCID for between-group comparisons, it remains clinically relevant for several reasons. First, the control group also exhibited natural recovery (within-group improvement of 21.50 m from T0 to T4), and the intervention group’s net gain still contributed to meaningful functional improvement at the individual level. Second, previous studies in CR have noted that even modest 6MWD improvements (30-45 m) are associated with better long-term prognosis [48,49]. Third, the intervention’s net effect was maintained across follow-up, indicating durable functional benefits. A systematic review found that the 6MWT could be used to determine clinical response to cardiac interventions and is likely a good marker of prognosis, and demonstrated an inverse relationship between 6MWD and the likelihood of a major adverse cardiac event (MACE) [48]. Moreover, a study demonstrated that each 50 m increase in 6MWT distance was associated with a lower odds ratio and hazard ratio for the MACE. The 6MWT has a prognostic value for predicting MACE in patients with prevalent CVDs [49]. Conversely, another researcher [50] reported that 6MWT may not be sensitive enough, unable to monitor early intervention for patients with normal functions. They indicated that familiarity with walking routes or a good walking pace could lead to an artificially increased walking distance. They suggested that a familiar experiment should be carried out, the second test to establish a baseline value. In future studies, we will consider multiple repeated measurements in more populations and incorporate familiarization tests into the 6MWT to further improve the accuracy of assessment and testing and to present more precise clinical value and significance.

A study by Cavalheri et al [51] has pointed out that fatigue is one of the most frequently cited barriers for patients to adhere to exercise programs, and that exercises or tools to alleviate fatigue symptoms are very important for patients and health care workers and should be the target of exercise interventions. Exercise interventions in populations, such as lung transplantation [52] and rheumatoid arthritis [53], have also been found to have a good effect on improving the degree of fatigue in patients. However, in a systematic review of the effects of exercise rehabilitation or exercise rehabilitation on health-related quality of life and fatigue in patients with lung cancer [54], none of the studies reported statistically significant reductions in fatigue. This may be due to the large heterogeneity of the physical exercise program, the short duration of the intervention in some studies, and the poor treatment effect in most studies. This may explain why self-perceived fatigue in this study did not improve significantly during the intervention period but decreased significantly during the follow-up period.

The exercise rehabilitation platform based on the PSD model significantly improves the ESE of patients after PCI during the intervention period and the follow-up period, and has a good maintenance effect. This is consistent with the intervention effect of ESE of patients in the sedentary behavior intervention program based on the behavior change wheel theory [55]. In the intervention process, the research participants are guided to actively participate in the change of sedentary behavior, and self-monitoring is carried out, so that their real sense is affected by the positive change after the interruption of sedentary behavior, and the self-efficacy, participation intention, and compliance of sedentary patients are improved. Alhasani et al [22] found that self-monitoring to support self-management of health problems related to the application is crucial; it enhances the user’s insight into their health, inspires them to seek medical help when necessary, and enhances patient confidence in the disease intervention process. In addition, the persuasive principle of praise is also selected, using incentives as a way of feedback to users. By evaluating the completion of exercise tasks, patients are sent praise messages, and certain points are rewarded, which will also strengthen the action of patients to take health behavior changes to a certain extent. Success depends on a digital self-management system related to health-promoting factors, including self-adjusting strategies, such as cognitive and emotional adjustment, goal setting, effective response, and problem-solving skills, and enhanced confidence in self-adjusting [56]. With time, changing the established behaviors and habits may be challenging, but in the future, eHealth interventions, combining health behaviors with persuasive strategies, may contribute to a sustainable behavior change in health management.

In Europe, especially Nordic countries, physiotherapists have long been core leaders in CR—driving clinical practice, program innovation, and research as key members of multidisciplinary teams, a role rooted in regional health care traditions [57]. In China, physiotherapists’ involvement in CR is evolving, but is more focused on clinical execution rather than leadership or research. This is partly due to a shortage of specialized rehabilitation professionals and underdeveloped training systems, with CR programs still predominantly being physician-led [58]. This difference reflects varied health care models. Nordic systems prioritize physiotherapists as CR innovators, while China’s developing CR infrastructure currently positions them as implementers—highlighting a need for workforce development to fully leverage their expertise.

### Limitations

This study has limitations that should be considered. First, participants were recruited from a single tertiary hospital in Hangzhou, which may limit the generalizability of findings to patients from primary or secondary hospitals or rural areas. Second, PA was assessed by the self-reported IPAQ, and while outcome assessors were blinded to group assignments, participants knew their group allocation due to the unblinded exercise intervention. This awareness may have influenced self-reporting (eg, overestimation or underestimation of activity), a bias that cannot be fully eliminated by only blinding data collectors [59-61]. Third, the study design compared “standard CR + eHealth platform” versus “standard CR alone” and thus cannot evaluate the standalone effectiveness of the eHealth platform. Fourth, the GEE model assumes that missing data are missing at random. However, some dropouts in the control group were due to myocardial infarction (a serious AE related to the underlying disease), which constitutes informative censoring (missing not at random). This assumption may introduce bias, as the reasons for dropout are associated with the outcome of interest (cardiac function and PA). Future studies could use sensitivity analyses (eg, inverse probability weighting) or alternative statistical models (eg, joint models for longitudinal data and survival) to account for informative censoring and enhance the robustness of the results. Other limitations include the application environment, cost budget, time, and the ability of the technical team [62], which also become the obstacle factors for the application of the WeChat mini program of exercise rehabilitation to a certain extent.

### Future Work

Future research directions should address these limitations. Priority could be given to direct comparative studies between center-based CR and the PSD model–based eHealth platform—a design that would clarify whether the eHealth tool can serve as a viable alternative for patients unable to attend in-person rehabilitation. Future studies could use objective tools (eg, accelerometers) to improve outcome reliability. Additionally, multicenter trials with larger sample sizes are needed to validate the effectiveness of the platform across diverse patient populations. Long-term follow-up should also be incorporated to assess the sustainability of outcomes, such as exercise adherence and quality of life.

### Conclusions

The findings of this study demonstrate the effectiveness of the eHealth CR platform based on the PSD model in improving key rehabilitation outcomes for patients after PCI. Specifically, when provided in addition to standard CR, the PSD model–based eHealth platform further enhanced patients’ PA level, exercise endurance, ESE, and quality of life, while also reducing self-perceived fatigue during the follow-up period. These findings provide practical insights for optimizing CR delivery in clinical settings. For health care providers, integrating PSD model–based eHealth tools into existing CR programs may serve as a feasible strategy to address suboptimal adherence and enhance long-term rehabilitation outcomes. For patients, the platform offers a flexible, accessible means to reinforce in-person rehabilitation guidance, particularly during postintervention follow-up.

## Appendix

Multimedia Appendix 1 Multi-dimensional Considerations in Exercise Prescription Library Construction.

Multimedia Appendix 2 CONSORT-eHEALTH checklist (V 1.6.1).

## Abbreviations

6MWD: 6-minute walk distance
6MWT: 6-minute walk test
AE: adverse event
BCT: behavior change technique
CHD: coronary heart disease
CONSORT: Consolidated Standards of Reporting Trials
CR: cardiac rehabilitation
CVD: cardiovascular disease
ESE: exercise self-efficacy
ESES: Exercise Self-Efficacy Scale
GEE: generalized estimating equation
IPAQ: International Physical Activity Questionnaire
MACE: major adverse cardiac event
MCID: minimum clinically important difference
MET: Metabolic Equivalent Task
mHealth: mobile health
PCI: percutaneous coronary intervention
PSD: persuasive systems design
PA: physical activity
RCR: remote cardiac rehabilitation
SUS: System Usability Scale
SF-36: Short Form of 36 Health Survey Questionnaire
SPO2: peripheral oxygen saturation
WHO: World Health Organization

## Data Availability

The datasets generated or analyzed during this study are available from the corresponding author (LH) on reasonable request.
