# Correction: Effects of an eHealth Cardiac Exercise Rehabilitation Platform for Patients After Percutaneous Coronary Intervention Based on the Persuasive Systems Design Model: Randomized Controlled Trial

**DOI:** 10.2196/99326

**Published:** 2026-05-05

**Authors:** Yang Liu, Xiting Huang, Ziying Dai, Zhili Jiang, Wenxiao Wu, Jing Wang, Zhiqian Wang, Luyao Yu, Hanyu Li, Lihua Huang

**Affiliations:** 1 Department of Nursing The First Affiliated Hospital Zhejiang University School of Medicine Hangzhou China; 2 Department of Nursing, Sun Yat-sen University Cancer Center Guangzhou China; 3 School of Humanities and Management, Zhejiang Chinese Medical University Hangzhou China

The authors noted two errors in “Effects of an eHealth Cardiac Exercise Rehabilitation Platform for Patients After Percutaneous Coronary Intervention Based on the Persuasive Systems Design Model: Randomized Controlled Trial” [1].

The first affiliation has been revised from the following:

The First Affiliated Hospital, Zhejiang University School of Medicine, Hangzhou, China

The affiliation now reads:

Department of Nursing, The First Affiliated Hospital, Zhejiang University School of Medicine, Hangzhou, China

The degrees of authors were originally listed as the following:

Yang Liu, MS; Xiting Huang, MS; Ziying Dai, MS; Zhili Jiang, MS; Wenxiao Wu, MS; Jing Wang, MS; Zhiqian Wang, MS; Luyao Yu, MS; Hanyu Li, MS; Lihua Huang, PhD

The list of degrees now reads:

Yang Liu, MSN; Xiting Huang, MM; Ziying Dai, MM; Zhili Jiang, MSN; Wenxiao Wu, MSN; Jing Wang, MSN; Zhiqian Wang, MSN; Luyao Yu, MSN; Hanyu Li, MSN; Lihua Huang, MBBS

These correction does not affect the scientific conclusions or validity of the original article.

The correction will appear in the online version of the paper on the JMIR Publications website, together with the publication of this correction notice. Because this was made after submission to PubMed, PubMed Central, and other full-text repositories, the corrected article has also been resubmitted to those repositories.
