# Supplementary material for: Effects of an eHealth Cardiac Exercise Rehabilitation Platform for Patients After Percutaneous Coronary Intervention Based on the Persuasive Systems Design Model: Randomized Controlled Trial
Source: J Med Internet Res. 2026 Jan 14;28:e71450. doi: 10.2196/71450 (PMC12853092; doi:10.2196/71450)
Supplement: Multimedia Appendix 1 [file jmir_v28i1e71450_app1.docx]

**Multi-dimensional Considerations in Exercise Prescription Library Construction**

The prescription library was refined by integrating three critical factors:

1. Environmental implementation feasibility

Considering that most patients would perform exercises at home (rather than in hospital settings), we excluded equipment-dependent activities (e.g., treadmill running) and prioritized exercises requiring minimal or no equipment:

Aerobic exercises: Brisk walking, stair climbing, stationary cycling (if available);

Resistance training: Bodyweight squats, wall push-ups, elastic band exercises (easily purchasable in local markets).

1. Safety safeguards

To minimize cardiovascular risks, each prescription included:

Contraindication screening: Exclusion of exercises triggering angina, severe arrhythmia, or blood pressure >180/110 mmHg (based on ATS guidelines);

Warm-up/cool-down protocols: Mandatory 5-minute warm-up (e.g., slow walking, joint mobility exercises) and 5-minute cool-down (e.g., stretching) to prevent sudden cardiac load changes;

Intensity ceiling: Maximum heart rate (HRmax) capped at 70% of age-predicted HRmax (220-age) for all patients, with lower thresholds (60% HRmax) for those with left ventricular ejection fraction <40%.

1. Individualization basis

The library was stratified into 3 tiers based on baseline 6-minute walk distance (6MWD) to ensure suitability for varying fitness levels:

Tier 1 (6MWD < 300m): Low-intensity (3.0-3.5 METs), short-duration (20-25 minutes/session) exercises;

Tier 2 (6MWD 300-450m): Moderate-intensity (3.5-4.5 METs), medium-duration (25-35 minutes/session) exercises;

Tier 3 (6MWD > 450m): Moderate-to-high intensity (4.5-5.0 METs), longer-duration (35-40 minutes/session) exercises.

**Specific Contents of the Prescription Library**

As shown in Fig. 2, the final library included 12 standardized prescriptions (4 per tier), each detailing:

Exercise type (e.g., "brisk walking + elastic band bicep curls");

FITT parameters: Frequency (e.g., "5 sessions/week"), Intensity (e.g., "3.5 METs, 60-65% HRmax"), Time (e.g., "30 minutes/session"), Type;

Progression criteria: When patients completed 3 consecutive sessions with heart rate <60% HRmax and no discomfort, they advanced to the next tier;

Safety alerts: Warning signs to stop exercise (e.g., chest pain, severe dyspnea) and emergency contact information.
